# Epidemiological and clinical course of 483 patients with COVID-19 in Wuhan, China: a single-center, retrospective study from the mobile cabin hospital

**DOI:** 10.1007/s10096-020-03927-3

**Published:** 2020-07-18

**Authors:** Bo Wang, Zhixian Wang, Jianping Zhao, Xiaoyong Zeng, Mingfu Wu, Shixuan Wang, Tiejun Wang

**Affiliations:** 1 Department of Obstetrics and Gynecology, Tongji Hospital, Tongji Medical College, Huazhong University of Science and Technology, Wuhan, 430030 China; 2 Department of Urology, Tongji Hospital, Tongji Medical College, Huazhong University of Science and Technology, Wuhan, 430030 China; 3 Department of Respiratory and Critical Care Medicine, National Clinical Research, Key Laboratory of Pulmonary Diseases of Health Ministry, Tongji Hospital, Tongji Medical College, Huazhong University of Science and Technology, Wuhan, 430030 Hubei China; 4 Department of Breast Surgery, Hubei Cancer Hospital, 116 Zhuodaoquan South Road, Wuhan, 430079 China

**Keywords:** COVID-19, characteristics, outcomes, mobile cabin hospital

## Abstract

During the COVID-19 outbreak, the mobile cabin hospital has effectively isolated and treated patients diagnosed as mild-moderate disease. However, a detailed clinical course has not been well described. We included 483 patients who were isolated and treated from Feb 6, 2020, to Feb 15, 2020, including definite outcome (discharge or deterioration). Sixty-two patients were transferred to severe cases, of whom were trasfered to designated hospital for intensive care. By March 9, 2020, all patients were discharged without dead. The mobile cabin hospital provides feasible strategy of isolation of mild-moderate cases and timely intervention during the virus outbreak.

**Electronic supplementary material:**

The online version of this article (10.1007/s10096-020-03927-3) contains supplementary material, which is available to authorized users.

## Introduction

**Fig. 1:**
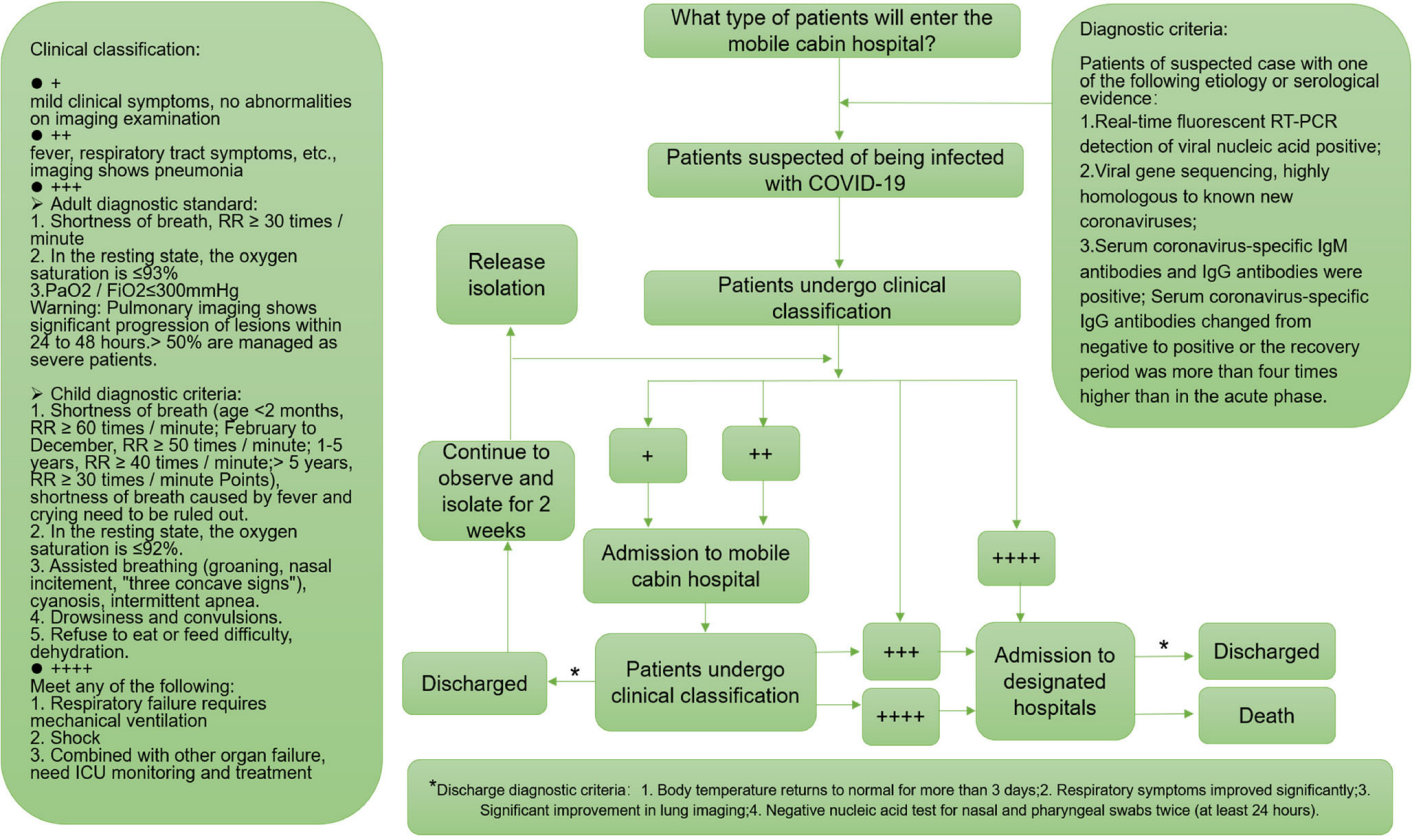
The flow chart of consultation for patients infected with COVID-19

Since December 8, 2019, Wuhan, Hubei, China, has reported several cases of COVID-19. In addition to China, other countries including South Korea, Iran, and Italy also have reported cases of COVID-19 infection [1, 2]. According to the “New Coronavirus Infected Pneumonia Diagnosis and Treatment Plan (Trial Version 5),” during the study period [3], severe and critically ill patients are at risk for secondary systemic multiple organ failure, which in turn increases the risk of death. Therefore, it is necessary to treat critically ill patients and also prevent mild-moderate cases from developing into severe cases. The mobile cabin hospital has played an important role in stemming China’s outbreak of COVID-19 infection, especially in isolating and treating patients diagnosed as mild-moderate disease. However, information about these patient’s characteristics and the outcomes are scarce. Although previous studies reported the clinical characteristics of patients with COVID-19 pneumonia [4–7], limited research focused on the patients who developed from mild-moderate to severe disease, our study mainly analyzed the clinical characteristics of these cases admitted to the mobile cabin hospital (Fig. 1).

## Results

Among the cohort of 483 patients, 62 patients (12.8%) progressed to severe cases, and 421 patients (87.2%) were cured. The median age was 50 years, and 54.9% of cases were female. Besides, 61.7% of patients were exposed to the suspected/confirmed patients, and 45.5% of patients experienced family cluster infection. We found increasing odds of severe cases associated with comorbidities, including primary pulmonary disease (7.6% vs. 16.1%, *p* = 0.047), coronary heart disease (0.7% vs. 8.1%, *p* < 0.001), and abnormal laboratory test of renal (0.2%vs. 16.1%, *p* < 0.001), liver function (1.9%vs. 19.4%, *p* < 0.001), renal function (0.2% vs. 16.1%, *p* < 0.001), heart function (1.2% vs. 19.4%, *p* < 0.001), and abnormal lymphocyte (15.2% vs. 37.1%, *p* < 0.001) and leukocyte counts (23.0% vs. 37.1%, *p* = 0.025) (Table 1). By March 9, 2020, all patients were as follows: 62 patients who were diagnosed as severe cases were transferred to a designated hospital for intensive care, of whom, 23 refused (alive confirmed), 8 patients were cured and discharged, and 31 patients were still in the designated hospital to continue treatment and recovered.

**Table 1:** Clinical characteristics of patients with coronavirus disease 2019

	Overall	Mild-moderate cases (+/++)	Severe cases (+++/++++)	*p*^a^ value
	(*n* = 483)	(*n* = 421)	(*n* = 62)	
Time from symptom to admission				0.585
Mean (SD, min, max)	5.86 (5.23, 0.00, 30.0)	5.90 (5.27, 0.00, 30.0)	5.52 (4.91, 0.00, 18.0)	
Median (IQR)	4.00 (2.00, 8.00)	4.00 (2.00, 8.00)	3.00 (2.00, 9.00)	
Time from admission to cure/severe illness				< 0.001
Mean (SD, min, max)	12.2 (4.71, 1.00, 23.0)	12.5 (4.51, 1.00, 23.0)	9.74 (5.32, 1.00, 23.0)	
Median (IQR)	12.0 (9.00, 15.0)	13.0 (9.00, 16.0)	9.00 (5.25, 13.8)	
Time from symptom to cure/severe illness				0.002
Mean (SD, min, max)	18.0 (7.41, 1.00, 50.0)	18.4 (7.35, 1.00, 50.0)	15.3 (7.32, 1.00, 50.0)	
Median (IQR)	17.0 (13.0, 23.0)	17.0 (13.0, 23.0)	14.5 (9.25, 21.0)	
Demographic characteristics				0.830
Age				
Mean (SD, min, max)	48.4 (12.4, 11.0, 83.0)	48.5 (12.4, 11.0, 72.0)	48.1 (12.9, 22.0, 83.0)	
Median (IQR)	50.0 (39.0, 58.0)	50.0 (39.0, 58.0)	48.5 (37.3, 58.8)	
Age distribution				0.360
(~ 50]	227 (47.0%)	194 (46.1%)	33 (53.2%)	
(50~	256 (53.0%)	227 (53.9%)	29 (46.8%)	
Sex				0.341
Female	265 (54.9%)	227 (53.9%)	38 (61.3%)	
Male	218 (45.1%)	194 (46.1%)	24 (38.7%)	
BMI				0.738
Mean (SD, min, max)	23.3 (3.15, 15.0, 37.8)	23.3 (3.15, 15.0, 37.8)	23.4 (3.15, 16.5, 31.3)	
Median (IQR)	22.9 (21.4, 25.4)	22.9 (21.4, 25.4)	23.1 (21.9, 25.5)	
BMI distribution				0.485
(~ 18.4]	25 (5.2%)	20 (4.8%)	5 (8.1%)	
(18.5, 23.9]	271 (56.1%)	239 (56.8%)	32 (51.6%)	
(24, 27.9]	155 (32.1%)	136 (32.3%)	19 (30.6%)	
[28~)	32 (6.6%)	26 (6.2%)	6 (9.7%)	
Huanan seafood wholesale market exposure				0.574
No	475 (98.3%)	413 (98.1%)	62 (100%)	
Yes	8 (1.7%)	8 (1.9%)	0 (0%)	
History suspected patient exposure				0.080
Uncertain	185 (38.3%)	168 (39.9%)	17 (27.4%)	
Yes	298 (61.7%)	253 (60.1%)	45 (72.6%)	
With other family member infected				0.011
No	263 (54.5%)	239 (56.8%)	24 (38.7%)	
Yes	220 (45.5%)	182 (43.2%)	38 (61.3%)	
Daily exercise				0.730
No	189 (39.1%)	163 (38.7%)	26 (41.9%)	
Yes	294 (60.9%)	258 (61.3%)	36 (58.1%)	
Daily self-care ability				< 0.001
No	22 (4.6%)	8 (1.9%)	14 (22.6%)	
Yes	461 (95.4%)	413 (98.1%)	48 (77.4%)	
Smoking				0.063
Current smokers	83 (17.2%)	66 (15.7%)	17 (27.4%)	
Give up smoking	15 (3.1%)	14 (3.3%)	1 (1.6%)	
Never smokers	385 (79.7%)	341 (81.0%)	44 (71.0%)	
Marital status				0.782
Divorce	3 (0.6%)	3 (0.7%)	0 (0%)	
Married	454 (94.0%)	395 (93.8%)	59 (95.2%)	
Unmarried	26 (5.4%)	23 (5.5%)	3 (4.8%)	
Recent surgery history				< 0.001
No	101 (20.9%)	99 (23.5%)	2 (3.2%)	
Yes	382 (79.1%)	322 (76.5%)	60 (96.8%)	
Antiviral drug treatment before admission				0.874
No	164 (34.0%)	144 (34.2%)	20 (32.3%)	
Yes	319 (66.0%)	277 (65.8%)	42 (67.7%)	
Antibiotic drug treatment before admission				0.723
No	193 (40.0%)	170 (40.4%)	23 (37.1%)	
Yes	290 (60.0%)	251 (59.6%)	39 (62.9%)	
Comorbidities at admission				
Primary pulmonary disease				0.047
No	441 (91.3%)	389 (92.4%)	52 (83.9%)	
Yes	42 (8.7%)	32 (7.6%)	10 (16.1%)	
Hypertension				0.067
No	449 (93.0%)	392 (93.1%)	57 (91.9%)	
Yes	34 (7.0%)	29 (6.9%)	5 (8.1%)	
Diabetes				0.072
No	459 (95.0%)	401 (95.2%)	58 (93.5%)	
Yes	24 (5.0%)	20 (4.8%)	4 (6.5%)	
Hyperlipidemia				0.124
No	470 (97.3%)	412 (97.9%)	58 (93.5%)	
Yes	13 (2.7%)	9 (2.1%)	4 (6.5%)	
Coronary heart disease				< 0.001
No	475 (98.3%)	418 (99.3%)	57 (91.9%)	
Yes	8 (1.7%)	3 (0.7%)	5 (8.1%)	
History of myocardial infarction				0.048
No	476 (98.6%)	417 (99.0%)	59 (95.2%)	
Yes	7 (1.4%)	4 (1.0%)	3 (4.8%)	
Cerebral infarction				0.849
No	478 (99.0%)	416 (98.8%)	62 (100%)	
Yes	5 (1.0%)	5 (1.2%)	0 (0%)	
Cerebral hemorrhage				0.266
No	482 (99.8%)	420 (99.8%)	62 (100%)	
Yes	1 (0.2%)	1 (0.2%)	0 (0%)	
Malignant neoplasms				0.849
No	478 (99.0%)	416 (98.8%)	62 (100%)	
Yes	5 (1.0%)	5 (1.2%)	0 (0%)	
Other disease				0.039
No	442 (91.5%)	390 (92.6%)	52 (83.9%)	
Yes	41 (8.5%)	31 (7.4%)	10 (16.1%)	
Vital signs at admission^b^				
Blood pressure				0.943
Hight blood pressure	34 (7.0%)	29 (6.9%)	5 (8.1%)	
Normal blood pressure	449 (93.0%)	392 (93.1%)	57 (91.9%)	
Breath				< 0.001
Breathing faster	32 (6.6%)	20 (4.8%)	12 (19.4%)	
Normal breathing	451 (93.4%)	401 (95.2%)	50 (80.6%)	
Heart rate				< 0.001
Increased heart rate	32 (6.6%)	21 (5.0%)	11 (17.7%)	
Normal heart rate	451 (93.4%)	400 (95.0%)	51 (82.3%)	
Symptom at admission				0.897
Mild	45 (9.3%)	39 (9.3%)	6 (9.7%)	
Moderate	438 (90.7%)	382 (90.7%)	56 (90.3%)	
Highest temperature				0.029
Mean (SD, min, max)	37.7 (0.86, 36.0, 40.0)	37.7 (0.86, 36.0, 40.0)	37.9 (0.89, 36.5, 39.9)	
Median (IQR)	37.8 (36.9, 38.4)	37.7 (36.8, 38.3)	37.9 (37.2, 38.6)	
Temperature distribution				0.195
< 37.5 °C	176 (36.4%)	158 (37.5%)	18 (29.0%)	
37.5–38.0 °C	112 (23.2%)	97 (23.0%)	15 (24.2%)	
38.1–39.0 °C	137 (28.4%)	113 (26.8%)	24 (38.7%)	
> 39.0 °C	58 (12.0%)	53 (12.6%)	5 (8.1%)	
Cough				0.524
No	204 (42.2%)	175 (41.6%)	29 (46.8%)	
Yes	279 (57.8%)	246 (58.4%)	33 (53.2%)	
Shortness of breath				0.041
No	352 (72.9%)	314 (74.6%)	38 (61.3%)	
Yes	131 (27.1%)	107 (25.4%)	24 (38.7%)	
Myalgia				0.439
No	334 (69.2%)	288 (68.4%)	46 (74.2%)	
Yes	149 (30.8%)	133 (31.6%)	16 (25.8%)	
Running nose				0.240
No	408 (84.5%)	352 (83.6%)	56 (90.3%)	
Yes	75 (15.5%)	69 (16.4%)	6 (9.7%)	
Arthralgia				0.263
No	383 (79.3%)	330 (78.4%)	53 (85.5%)	
Yes	100 (20.7%)	91 (21.6%)	9 (14.5%)	
Chest tightness				0.288
No	365 (75.6%)	322 (76.5%)	43 (69.4%)	
Yes	118 (24.4%)	99 (23.5%)	19 (30.6%)	
Nausea or vomiting				0.015
No	415 (85.9%)	355 (84.3%)	60 (96.8%)	
Yes	68 (14.1%)	66 (15.7%)	2 (3.2%)	
Headache				0.892
No	381 (78.9%)	333 (79.1%)	48 (77.4%)	
Yes	102 (21.1%)	88 (20.9%)	14 (22.6%)	
Fatigue				0.833
No	461 (95.4%)	401 (95.2%)	60 (96.8%)	
Yes	22 (4.6%)	20 (4.8%)	2 (3.2%)	
Pharyngalgia				0.606
No	481 (99.6%)	419 (99.5%)	62 (100%)	
Yes	2 (0.4%)	2 (0.5%)	0 (0%)	
Nasal congestion				0.606
No	481 (99.6%)	419 (99.5%)	62 (100%)	
Yes	2 (0.4%)	2 (0.5%)	0 (0%)	
Diarrhea				0.012
No	447 (92.5%)	395 (93.8%)	52 (83.9%)	
Yes	36 (7.5%)	26 (6.2%)	10 (16.1%)	
Chill				
No	478 (99.0%)	417 (99.0%)	61 (98.4%)	
Yes	5 (1.0%)	4 (1.0%)	1 (1.6%)	
Laboratory test results at admission ^c^				
Leukocyte				0.025
Abnormal	120 (24.8%)	97 (23.0%)	23 (37.1%)	
Normal	363 (75.2%)	324 (77.0%)	39 (62.9%)	
Lymphocyte				< 0.001
Abnormal	87 (18.0%)	64 (15.2%)	23 (37.1%)	
Normal	396 (82.0%)	357 (84.8%)	39 (62.9%)	
Blood glucose				0.374
Abnormal glucose	24 (5.0%)	19 (4.5%)	5 (8.1%)	
Normal glucose	459 (95.0%)	402 (95.5%)	57 (91.9%)	
Renal function				< 0.001
Normal	472 (97.7%)	420 (99.8%)	52 (83.9%)	
Abnormal	11 (2.3%)	1 (0.2%)	10 (16.1%)	
Heart function				< 0.001
Normal	466 (96.5%)	416 (98.8%)	50 (80.6%)	
Abnormal	17 (3.5%)	5 (1.2%)	12 (19.4%)	
Liver function				< 0.001
Normal	463 (95.9%)	413 (98.1%)	50 (80.6%)	
Abnormal	20 (4.1%)	8 (1.9%)	12 (19.4%)	
Urine infection				0.129
No	435 (90.1%)	383 (91.0%)	52 (83.9%)	
Yes	48 (9.9%)	38 (9.0%)	10 (16.1%)	
Imaging of lung				< 0.001
Normal	458 (94.8%)	415 (98.6%)	43 (69.4%)	
Abnormal	25 (5.2%)	6 (1.4%)	19 (30.6%)	
Mental state before admission^d^				0.076
Nervous before admission	166 (34.4%)	138 (32.8%)	28 (45.2%)	
Without nervous before admission	317 (65.6%)	283 (67.2%)	34 (54.8%)	
Sleep quality since diagnosis				0.005
Bad	123 (25.5%)	97 (23.0%)	26 (41.9%)	
Good	20 (4.1%)	19 (4.5%)	1 (1.6%)	
Without influence	340 (70.4%)	305 (72.4%)	35 (56.5%)	

^a^Data are *n* (%) unless otherwise specified; *p* values demonstrate differences between No conversion to severe and conversion to severe patients. *p* < 0.05 was considered obviously significant

^b^Hypertension, ≥ 140/90 mmHg; breath, 12–20 times/min; heart rate, 60–100 times/min

^c^Normal reference value [1]: leukocyte: adult, (4.0–10.0) × 10^9/L; child, (5.0–12.0) × 10^9/L [2]; lymphocyte percentage (Lymph%) 20–40%; lymphocyte absolute value (Lymph #) 1.1–3.2 × 10^9 [3]; fasting whole blood glucose 3.9~6.1 mmol/L, 1 h after meal 6.7~9.4 mmol/L, 2 h after meal ≤ 7.8 mmol/L

^d^Heart function: tachycardia (100 beats/min)

^e^Liver function: ALT 0–46 U/L; AST 0–46 U/L

^f^Urine infection: creatinine (30–110 umol/L)

## Discussion

During the COVID-19 outbreak, the number of confirmed cases has exploded in China. The major challenge is to treat and isolate these patients, as well as reduce severe cases and mortality. The establishment of the mobile cabin hospital has witnessed the classification management effectively. In this study, all patients received a nucleic acid test before admission; after the patients were admitted to the mobile cabin hospital, the treatment was carried out according to the “New Coronavirus Infected Pneumonia Diagnosis and Treatment Plan” [3]. To our knowlegement, this is the largest retrospective cohort study among mild-moderate cases with COVID-19 infection; the clinical course with respect to mild-moderate and severe cases in Wuchang mobile cabin hospital were analyzed in this study.

Our results showed that there was no significant difference in fever between mild-moderate and severe cases, of whom 421 (87.2%) patients were not admitted to the ICU, and 263 (62.5%) patients were identified as having a fever but progressed to critically ill status, suggesting that there may be individual differences in body temperature monitoring and even in the early concealment of the virus [8]. Consistent with the transmission route, we also found that critically ill patients were characterized by familial cluster infections, which indirectly confirms that COVID-19 can be transmitted through contact [9–11]. If necessary, appropriate psychological intervention during the admission of a patient may contribute to elevating the patient’s condition.

So far, the COVID-19 infection has been managed by controlling the source of infection and cutting off the route of transmission dominates, but no effective treatment has been proposed. For critically ill patients, supportive treatments may continue for some time. According to our study, all the cases in the mobile cabin hospital were community-acquired viral infections; no cases of nosocomial infections were found. This also suggested that the safety isolation measures adopted by patients and medical workers in the mobile cabin hospital can significantly reduce the chance of cross-infection.

Several limitations should be highlighted. First, this was a retrospective study and inherent limitations existed; we tried our best to collect detailed information, but not all laboratory information were collected adequately. Second, 23 patients were lost to follow-up, including 10 of them who also refused our follow-up (for this part, the medical records suggested that they were alive), which enabled a lack of analyzing the outcome of patients after being transferred to designated hospital. These patients who lost to follow-up may have a certain impact on the results, especially deviations and existing biases, so exploring the potential risks associated with the deterioration of patients is infeasible. However, depending on this descriptive study, we found that severe cases were associated with comorbidities. We believe that our study population is representative of mild-moderate cases, especially those who transferred to severe cases, for which provided feasible tactics in management of COVID-19 infection.

## Electronic supplementary material

ESM 1 (DOCX 786 kb)

ESM 2 (DOCX 40 kb)

## Abbreviations

BMI: Body mass index
IQR: Interquartile range
SD: Standard deviation
COVID-19: Corona virus disease 2019
