# Supplementary figures and images for: Epidemiological and clinical course of 483 patients with COVID-19 in Wuhan, China: a single-center, retrospective study from the mobile cabin hospital
Source: Eur J Clin Microbiol Infect Dis. 2020 Jul 18;39(12):2309–15. doi: 10.1007/s10096-020-03927-3 (PMC7368625; doi:10.1007/s10096-020-03927-3)

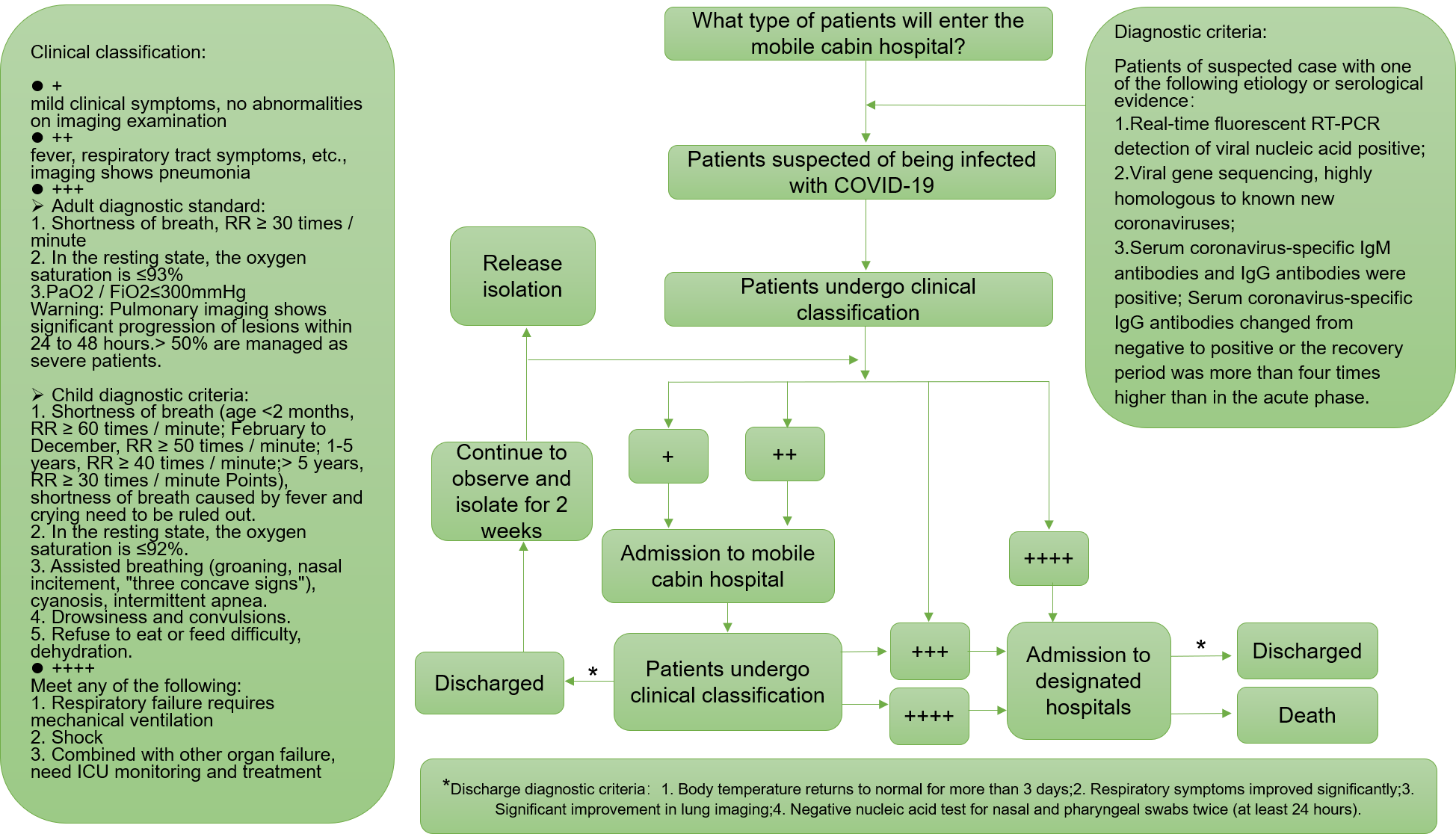


Figures1.The flow chart of consultation for patients infected with COVID-19

Supplement: Supplementary file 1 — (DOCX 786 kb) [file 10096_2020_3927_MOESM1_ESM.docx]
