# Supplementary material for: Epidemiological and clinical course of 483 patients with COVID-19 in Wuhan, China: a single-center, retrospective study from the mobile cabin hospital
Source: Eur J Clin Microbiol Infect Dis. 2020 Jul 18;39(12):2309–15. doi: 10.1007/s10096-020-03927-3 (PMC7368625; doi:10.1007/s10096-020-03927-3)
Supplement: Supplementary file 2 — (DOCX 40 kb) [file 10096_2020_3927_MOESM2_ESM.docx]

| Table 1. Clinical characteristics of patients with Coronavirus Disease 2019 | | | | |
| --- | --- | --- | --- | --- |
|  | **Overall** | **Mild (+)** | **Severe (++)** | ***p*^a^*-*value** |
|  | **(n=483)** | **(n=421)** | **(n=62)** |  |
| **Admission time** |  |  |  | - |
| 6-Feb-20 | 158 (32.7%) | 142 (33.7%) | 16 (25.8%) |  |
| 7-Feb-20 | 74 (15.3%) | 59 (14.0%) | 15 (24.2%) |  |
| 8-Feb-20 | 32 (6.6%) | 30 (7.1%) | 2 (3.2%) |  |
| 9-Feb-20 | 25 (5.2%) | 21 (5.0%) | 4 (6.5%) |  |
| 10-Feb-20 | 17 (3.5%) | 15 (3.6%) | 2 (3.2%) |  |
| 11-Feb-20 | 23 (4.8%) | 20 (4.8%) | 3 (4.8%) |  |
| 12-Feb-20 | 30 (6.2%) | 29 (6.9%) | 1 (1.6%) |  |
| 13-Feb-20 | 54 (11.2%) | 50 (11.9%) | 4 (6.5%) |  |
| 14-Feb-20 | 35 (7.2%) | 29 (6.9%) | 6 (9.7%) |  |
| 15-Feb-20 | 35 (7.2%) | 26 (6.2%) | 9 (14.5%) |  |
| **Time from symptom to admission** |  |  |  | 0.585 |
| Mean (SD, Min, Max) | 5.86 (5.23,0.00, 30.0) | 5.90 (5.27,0.00, 30.0) | 5.52 (4.91,0.00, 18.0) |  |
| Median (IQR) | 4.00 (2.00, 8.00) | 4.00 (2.00, 8.00) | 3.00 (2.00, 9.00) |  |
| **Time from admission to cure/severe illness** |  |  |  | <0.001 |
| Mean (SD, Min, Max) | 12.2 (4.71,1.00, 23.0) | 12.5 (4.51,1.00, 23.0) | 9.74 (5.32,1.00, 23.0) |  |
| Median (IQR) | 12.0 (9.00, 15.0) | 13.0 (9.00, 16.0) | 9.00 (5.25, 13.8) |  |
| **Time from symptom to cure/severe illness** |  |  |  | 0.002 |
| Mean (SD, Min, Max) | 18.0 (7.41,1.00, 50.0) | 18.4 (7.35,1.00, 50.0) | 15.3 (7.32,1.00, 50.0) |  |
| Median (IQR) | 17.0 (13.0, 23.0) | 17.0 (13.0, 23.0) | 14.5 (9.25, 21.0) |  |
| **Demographic characteristics** |  |  |  | 0.830 |
| **Age** |  |  |  |  |
| Mean (SD, Min, Max) | 48.4 (12.4,11.0, 83.0) | 48.5 (12.4,11.0, 72.0) | 48.1 (12.9,22.0, 83.0) |  |
| Median (IQR) | 50.0 (39.0, 58.0) | 50.0 (39.0, 58.0) | 48.5 (37.3, 58.8) |  |
| **Age distribution** |  |  |  | 0.360 |
| (~50] | 227 (47.0%) | 194 (46.1%) | 33 (53.2%) |  |
| (50~ | 256 (53.0%) | 227 (53.9%) | 29 (46.8%) |  |
| **Sex** |  |  |  | 0.341 |
| Female | 265 (54.9%) | 227 (53.9%) | 38 (61.3%) |  |
| Male | 218 (45.1%) | 194 (46.1%) | 24 (38.7%) |  |
| **BMI** |  |  |  | 0.738 |
| Mean (SD, Min, Max) | 23.3 (3.15,15.0, 37.8) | 23.3 (3.15,15.0, 37.8) | 23.4 (3.15,16.5, 31.3) |  |
| Median (IQR) | 22.9 (21.4, 25.4) | 22.9 (21.4, 25.4) | 23.1 (21.9, 25.5) |  |
| **BMI distribution** |  |  |  | 0.485 |
| (~18.4] | 25 (5.2%) | 20 (4.8%) | 5 (8.1%) |  |
| (18.5,23.9] | 271 (56.1%) | 239 (56.8%) | 32 (51.6%) |  |
| (24,27.9] | 155 (32.1%) | 136 (32.3%) | 19 (30.6%) |  |
| [28~) | 32 (6.6%) | 26 (6.2%) | 6 (9.7%) |  |
| **Huanan seafood wholesale market exposure** |  |  |  | 0.574 |
| No | 475 (98.3%) | 413 (98.1%) | 62 (100%) |  |
| Yes | 8 (1.7%) | 8 (1.9%) | 0 (0%) |  |
| **History suspected patient exposure** |  |  |  | 0.080 |
| Uncertain | 185 (38.3%) | 168 (39.9%) | 17 (27.4%) |  |
| Yes | 298 (61.7%) | 253 (60.1%) | 45 (72.6%) |  |
| **With other family member infected** |  |  |  | 0.011 |
| No | 263 (54.5%) | 239 (56.8%) | 24 (38.7%) |  |
| Yes | 220 (45.5%) | 182 (43.2%) | 38 (61.3%) |  |
| **Daily exercise** |  |  |  | 0.730 |
| No | 189 (39.1%) | 163 (38.7%) | 26 (41.9%) |  |
| Yes | 294 (60.9%) | 258 (61.3%) | 36 (58.1%) |  |
| **Daily self-care ability** |  |  |  | <0.001 |
| No | 22 (4.6%) | 8 (1.9%) | 14 (22.6%) |  |
| Yes | 461 (95.4%) | 413 (98.1%) | 48 (77.4%) |  |
| **Smoking** |  |  |  | 0.063 |
| Current smokers | 83 (17.2%) | 66 (15.7%) | 17 (27.4%) |  |
| give up smoking | 15 (3.1%) | 14 (3.3%) | 1 (1.6%) |  |
| Never smokers | 385 (79.7%) | 341 (81.0%) | 44 (71.0%) |  |
| **Marital status** |  |  |  | 0.782 |
| Divorce | 3 (0.6%) | 3 (0.7%) | 0 (0%) |  |
| Married | 454 (94.0%) | 395 (93.8%) | 59 (95.2%) |  |
| Unmarried | 26 (5.4%) | 23 (5.5%) | 3 (4.8%) |  |
| **Recent Surgery history** |  |  |  | <0.001 |
| No | 101 (20.9%) | 99 (23.5%) | 2 (3.2%) |  |
| Yes | 382 (79.1%) | 322 (76.5%) | 60 (96.8%) |  |
| **Antiviral drug treatment before admission** |  |  |  | 0.874 |
| No | 164 (34.0%) | 144 (34.2%) | 20 (32.3%) |  |
| Yes | 319 (66.0%) | 277 (65.8%) | 42 (67.7%) |  |
| **Antibiotic drug treatment before admission** |  |  |  | 0.723 |
| No | 193 (40.0%) | 170 (40.4%) | 23 (37.1%) |  |
| Yes | 290 (60.0%) | 251 (59.6%) | 39 (62.9%) |  |
| **Comorbidities at admission** |  |  |  |  |
| **Primary pulmonary disease** |  |  |  | 0.047 |
| No | 441 (91.3%) | 389 (92.4%) | 52 (83.9%) |  |
| Yes | 42 (8.7%) | 32 (7.6%) | 10 (16.1%) |  |
| **Hypertension** |  |  |  | 0.067 |
| No | 449 (93.0%) | 392 (93.1%) | 57 (91.9%) |  |
| Yes | 34 (7.0%) | 29 (6.9%) | 5 (8.1%) |  |
| **Diabetes** |  |  |  | 0.072 |
| No | 459 (95.0%) | 401 (95.2%) | 58 (93.5%) |  |
| Yes | 24 (5.0%) | 20 (4.8%) | 4 (6.5%) |  |
| **Hyperlipidemia** |  |  |  | 0.124 |
| No | 470 (97.3%) | 412 (97.9%) | 58 (93.5%) |  |
| Yes | 13 (2.7%) | 9 (2.1%) | 4 (6.5%) |  |
| **Coronary heart disease** |  |  |  | <0.001 |
| No | 475 (98.3%) | 418 (99.3%) | 57 (91.9%) |  |
| Yes | 8 (1.7%) | 3 (0.7%) | 5 (8.1%) |  |
| **History of myocardial infarction** |  |  |  | 0.048 |
| No | 476 (98.6%) | 417 (99.0%) | 59 (95.2%) |  |
| Yes | 7 (1.4%) | 4 (1.0%) | 3 (4.8%) |  |
| **Cerebral infarction** |  |  |  | 0.849 |
| No | 478 (99.0%) | 416 (98.8%) | 62 (100%) |  |
| Yes | 5 (1.0%) | 5 (1.2%) | 0 (0%) |  |
| **Cerebral hemorrhage** |  |  |  | 0.266 |
| No | 482 (99.8%) | 420 (99.8%) | 62 (100%) |  |
| Yes | 1 (0.2%) | 1 (0.2%) | 0 (0%) |  |
| **Malignant neoplasms** |  |  |  | 0.849 |
| No | 478 (99.0%) | 416 (98.8%) | 62 (100%) |  |
| Yes | 5 (1.0%) | 5 (1.2%) | 0 (0%) |  |
| **Other disease** |  |  |  | 0.039 |
| No | 442 (91.5%) | 390 (92.6%) | 52 (83.9%) |  |
| Yes | 41 (8.5%) | 31 (7.4%) | 10 (16.1%) |  |
| **Vital signs at admission ^b^** |  |  |  |  |
| **Blood pressure** |  |  |  | 0.943 |
| Hight blood pressure | 34 (7.0%) | 29 (6.9%) | 5 (8.1%) |  |
| Normal blood pressure | 449 (93.0%) | 392 (93.1%) | 57 (91.9%) |  |
| **Breath** |  |  |  | <0.001 |
| Breathing faster | 32 (6.6%) | 20 (4.8%) | 12 (19.4%) |  |
| Normal breathing | 451 (93.4%) | 401 (95.2%) | 50 (80.6%) |  |
| **Heart rate** |  |  |  | <0.001 |
| Increased heart rate | 32 (6.6%) | 21 (5.0%) | 11 (17.7%) |  |
| Normal heart rate | 451 (93.4%) | 400 (95.0%) | 51 (82.3%) |  |
| **Symptom at admission** |  |  |  | 0.897 |
| Mild | 45 (9.3%) | 39 (9.3%) | 6 (9.7%) |  |
| Moderate | 438 (90.7%) | 382 (90.7%) | 56 (90.3%) |  |
| **Highest temperature** |  |  |  | 0.029 |
| Mean (SD, Min, Max) | 37.7 (0.86,36.0, 40.0) | 37.7 (0.86,36.0, 40.0) | 37.9 (0.89,36.5, 39.9) |  |
| Median (IQR) | 37.8 (36.9, 38.4) | 37.7 (36.8, 38.3) | 37.9 (37.2, 38.6) |  |
| **Temperature distribution** |  |  |  | 0.195 |
| <37.5℃ | 176 (36.4%) | 158 (37.5%) | 18 (29.0%) |  |
| 37.5-38.0℃ | 112 (23.2%) | 97 (23.0%) | 15 (24.2%) |  |
| 38.1-39.0℃ | 137 (28.4%) | 113 (26.8%) | 24 (38.7%) |  |
| > 39.0℃ | 58 (12.0%) | 53 (12.6%) | 5 (8.1%) |  |
| **Cough** |  |  |  | 0.524 |
| No | 204 (42.2%) | 175 (41.6%) | 29 (46.8%) |  |
| Yes | 279 (57.8%) | 246 (58.4%) | 33 (53.2%) |  |
| **Shortness of breath** |  |  |  | 0.041 |
| No | 352 (72.9%) | 314 (74.6%) | 38 (61.3%) |  |
| Yes | 131 (27.1%) | 107 (25.4%) | 24 (38.7%) |  |
| **Myalgia** |  |  |  | 0.439 |
| No | 334 (69.2%) | 288 (68.4%) | 46 (74.2%) |  |
| Yes | 149 (30.8%) | 133 (31.6%) | 16 (25.8%) |  |
| **Running nose** |  |  |  | 0.240 |
| No | 408 (84.5%) | 352 (83.6%) | 56 (90.3%) |  |
| Yes | 75 (15.5%) | 69 (16.4%) | 6 (9.7%) |  |
| **Arthralgia** |  |  |  | 0.263 |
| No | 383 (79.3%) | 330 (78.4%) | 53 (85.5%) |  |
| Yes | 100 (20.7%) | 91 (21.6%) | 9 (14.5%) |  |
| **Chest tightness** |  |  |  | 0.288 |
| No | 365 (75.6%) | 322 (76.5%) | 43 (69.4%) |  |
| Yes | 118 (24.4%) | 99 (23.5%) | 19 (30.6%) |  |
| **Nausea or vomiting** |  |  |  | 0.015 |
| No | 415 (85.9%) | 355 (84.3%) | 60 (96.8%) |  |
| Yes | 68 (14.1%) | 66 (15.7%) | 2 (3.2%) |  |
| **Headache** |  |  |  | 0.892 |
| No | 381 (78.9%) | 333 (79.1%) | 48 (77.4%) |  |
| Yes | 102 (21.1%) | 88 (20.9%) | 14 (22.6%) |  |
| **Fatigue** |  |  |  | 0.833 |
| No | 461 (95.4%) | 401 (95.2%) | 60 (96.8%) |  |
| Yes | 22 (4.6%) | 20 (4.8%) | 2 (3.2%) |  |
| **Pharyngalgia** |  |  |  | 0.606 |
| No | 481 (99.6%) | 419 (99.5%) | 62 (100%) |  |
| Yes | 2 (0.4%) | 2 (0.5%) | 0 (0%) |  |
| **Nasal congestion** |  |  |  | 0.606 |
| No | 481 (99.6%) | 419 (99.5%) | 62 (100%) |  |
| Yes | 2 (0.4%) | 2 (0.5%) | 0 (0%) |  |
| **Diarrhea** |  |  |  | 0.012 |
| No | 447 (92.5%) | 395 (93.8%) | 52 (83.9%) |  |
| Yes | 36 (7.5%) | 26 (6.2%) | 10 (16.1%) |  |
| **Chill** |  |  |  |  |
| No | 478 (99.0%) | 417 (99.0%) | 61 (98.4%) |  |
| Yes | 5 (1.0%) | 4 (1.0%) | 1 (1.6%) |  |
| **Laboratory test results at admission ^c^** |  |  |  |  |
| **Leukocyte** |  |  |  | 0.025 |
| Abnormal | 120 (24.8%) | 97 (23.0%) | 23 (37.1%) |  |
| Normal | 363 (75.2%) | 324 (77.0%) | 39 (62.9%) |  |
| **Lymphocyte** |  |  |  | <0.001 |
| Abnormal | 87 (18.0%) | 64 (15.2%) | 23 (37.1%) |  |
| Normal | 396 (82.0%) | 357 (84.8%) | 39 (62.9%) |  |
| **Blood glucose** |  |  |  | 0.374 |
| abnormal glucose | 24 (5.0%) | 19 (4.5%) | 5 (8.1%) |  |
| Normal glucose | 459 (95.0%) | 402 (95.5%) | 57 (91.9%) |  |
| **Renal function** |  |  |  | <0.001 |
| Normal | 472 (97.7%) | 420 (99.8%) | 52 (83.9%) |  |
| Abnormal | 11 (2.3%) | 1 (0.2%) | 10 (16.1%) |  |
| **Heart function** |  |  |  | <0.001 |
| Normal | 466 (96.5%) | 416 (98.8%) | 50 (80.6%) |  |
| Abnormal | 17 (3.5%) | 5 (1.2%) | 12 (19.4%) |  |
| **Liver function** |  |  |  | <0.001 |
| Normal | 463 (95.9%) | 413 (98.1%) | 50 (80.6%) |  |
| Abnormal | 20 (4.1%) | 8 (1.9%) | 12 (19.4%) |  |
| **Urine infection** |  |  |  | 0.129 |
| No | 435 (90.1%) | 383 (91.0%) | 52 (83.9%) |  |
| Yes | 48 (9.9%) | 38 (9.0%) | 10 (16.1%) |  |
| **Imaging of lung** |  |  |  | <0.001 |
| Normal | 458 (94.8%) | 415 (98.6%) | 43 (69.4%) |  |
| Abnormal | 25 (5.2%) | 6 (1.4%) | 19 (30.6%) |  |
| **Mental state before admission ^d^** |  |  |  | 0.076 |
| Nervous before admission | 166 (34.4%) | 138 (32.8%) | 28 (45.2%) |  |
| Without nervous before admission | 317 (65.6%) | 283 (67.2%) | 34 (54.8%) |  |
| **Sleep quality since diagnosis** |  |  |  | 0.005 |
| Bad | 123 (25.5%) | 97 (23.0%) | 26 (41.9%) |  |
| Good | 20 (4.1%) | 19 (4.5%) | 1 (1.6%) |  |
| Without influence | 340 (70.4%) | 305 (72.4%) | 35 (56.5%) |  |

**Abbreviations:** BMI: Body Mass Index; IQR: interquartile range; SD: standard deviation; COVID-19: Corona Virus Disease 2019.

a. *Data are n (%) unless otherwise specified; *p* values demonstrate differences between No conversion to severe and conversion to severe patients. P<.05 was considered obviously significant.

b. Hypertension: ≥140 / 90mmHg; Breath: 12-20 times/minute; Heart rate:60-100 times/minute.

c. Normal reference value: (1) Leukocyte: Adult: (4.0-10.0) × 10 ^ 9 / L; Child: (5.0-12.0) × 10 ^ 9 / L; (2) Lymphocyte percentage (Lymph%) 20-40%;Lymphocyte absolute value (Lymph #) 1.1-3.2 × 10 ^ 9; (3) Fasting whole blood glucose: 3.9 ~6.1 mmol / L, 1 hour after meal: 6.7~9.4 mmol/L, 2 hours after meal: ≤7.8 mmol/L. d. Heart function: Tachycardia (100 beats / min). e. Liver function: ALT 0-46U/L; AST 0-46U/L. f. Urine infection: Creatinine (30-110 umol/L).
